# An entrepreneurial training model to enhance undergraduate training in biomedical research

**DOI:** 10.1186/s12919-017-0091-8

**Published:** 2017-12-04

**Authors:** Farin Kamangar, Gillian Silver, Christine Hohmann, Cleo Hughes-Darden, Jocelyn Turner-Musa, Robert Trent Haines, Avis Jackson, Nelson Aguila, Payam Sheikhattari

**Affiliations:** 1 ASCEND Center for Biomedical Research, Division of Research & Economic Development, Morgan State University, Baltimore, MD USA; 2 Department of Biology, School of Computer, Mathematical, and Natural Sciences, Morgan State University, Baltimore, MD USA; 3 Department of Psychology, College of Liberal Arts, Morgan State University, Baltimore, MD USA; 4 Center to Reduce Cancer Health Disparities, National Cancer Institute, National Institutes of Health, Bethesda, MD USA; 5 Department of Behavioral Health Sciences, School of Community Health and Policy, Morgan State University, Baltimore, MD USA

## Abstract

**Background:**

Undergraduate students who are interested in biomedical research typically work on a faculty member’s research project, conduct one distinct task (e.g., running gels), and, step by step, enhance their skills. This “apprenticeship” model has been helpful in training many distinguished scientists over the years, but it has several potential drawbacks. For example, the students have limited autonomy, and may not understand the big picture, which may result in students giving up on their goals for a research career. Also, the model is costly and may greatly depend on a single mentor.

**Key highlights:**

The NIH Building Infrastructure Leading to Diversity (BUILD) Initiative has been established to fund innovative undergraduate research training programs and support institutional and faculty development of the recipient university. The training model at Morgan State University (MSU), namely “**A S**tudent-**C**entered **En**trepreneurship **D**evelopment training model” (ASCEND), is one of the 10 NIH BUILD-funded programs, and offers a novel, experimental “entrepreneurial” training approach. In the ASCEND training model, the students take the lead. They own the research, understand the big picture, and experience the entire scope of the research process, which we hypothesize will lead to a greater sense of self-efficacy and research competency, as well as an enhanced sense of science identity. They are also immersed in environments with substantial peer support, where they can exchange research ideas and share experiences. This is important for underrepresented minority students who might have fewer role models and less peer support in conducting research.

**Implications:**

In this article, we describe the MSU ASCEND entrepreneurial training model’s components, rationale, and history, and how it may enhance undergraduate training in biomedical research that may be of benefit to other institutions. We also discuss evaluation methods, possible sustainability solutions, and programmatic challenges that can affect all types of science training interventions.

## Background and setting

The makeup of the United States biomedical research workforce does not reflect the diversity of the nation’s population. For example, while African Americans, Hispanics, Native Americans, and Hawaiians/Pacific Islanders comprise over 30% of the US population, they constitute less than 10% of the nation’s scientific research faculty positions [1]. Lower diversity may lead to perceptions of unfairness, as well as inadequate use of the nation’s intellectual capital [1]. A major leak in the educational pipeline occurs between undergraduate and graduate degrees. While the above-mentioned groups collectively earned 22% of US bachelor’s degrees in science and engineering in 2014, they earned only 13% of the doctoral degrees in these fields [2].

For decades, the National Institutes of Health (NIH) has funded various diversity-related programs, such as Maximizing Access to Research Careers (MARC) or the National Institute of General Medical Sciences (NIGMS) Research Initiative for Scientific Enhancement (RISE). These programs typically provide intense research mentoring to students in faculty-initiated research, fund students to complete their undergraduate degree, provide role models, facilitate taking the Graduate Record Exam (GRE) and other tests necessary to be admitted to graduate school, and provide travel funds to national research meetings, among other things. While these programs have been very successful in training some distinguished researchers, there are still several challenges to achieve a substantial and sustainable increase in diversity [3]. Therefore, NIH postulated that other elements are needed to enhance these training programs.

In 2014, NIH announced the Building Infrastructure Leading to Diversity (BUILD) Initiative, [4] with the aim of diversifying the biomedical research workforce, focusing on undergraduate students. In response, Morgan State University (MSU), a historically black university in Baltimore, Maryland, proposed a novel model for training undergraduate students and was selected as one of the award recipients.

MSU is currently a university with approximately 7700 undergraduate and graduate students. While the university has always served all races and ethnic groups, the large majority of students are African-American and from underserved backgrounds; close to 70% are Pell Grant recipients. A large percentage of students are first-generation college students, who may not have strong support networks, and are at high risk of dropping out. Therefore, like other Historically Black Colleges and Universities (HBCUs), an important mission remains providing excellent training and a supportive environment. In addition, MSU has made major efforts to increase its status to a doctoral granting research university. In 1988, the state designated the campus as Maryland’s Public Urban University [5]. The composition of the university students, as well as its research mission, make it a good place to study educational interventions that are aimed to increase the diversity of the research workforce.

The training program offered at MSU is named ASCEND (**A S**tudent-**C**entered **En**trepreneurship **D**evelopment training model), and its main aim is to offer the useful elements of previous training programs (e.g., appropriate mentoring and financial assistance), with substantially enhanced peer support, [6] sense of science identity, [7] research competency, [7] and self-efficacy, [8–10] via several interventions. Most importantly, the students, with the support of the faculty, take leading roles in research by proposing research topics, writing proposals, conducting studies, analyzing data, and disseminating results.

The purpose of this paper is to discuss the ASCEND training model’s rationale and history, describe its main interventions, provide a framework for evaluation, and discuss challenges and lessons learned.

## Concept and rationale for the ASCEND entrepreneurial training model

The concept of ASCEND has roots in the Alma-Ata Declaration of 1978, a major milestone in the field of public health, which identified primary health care as the key to reaching the goal of “Health for All” [11]. To enhance primary health care research, particularly in low-resource and remote areas of the world, the World Health Organization offered health administrators and health workers short-term (one- to three-week) workshops, on how to select a research topic, how to write a proposal, and how to collect data. The workshops were followed by several months of data collection, and then another workshop on statistical analysis and interpretation of data. The underlying assumption was that the local health administrators and health workers would know of and choose to work on problems that are prevalent and important in their region. Given that they were the owners of the research, they persevered and were often able to collect the data, despite the fact that they were not professional researchers. (ASCEND’s Summer Research Institute, described below, uses a similar model.)

In the early 1990s, Iranian medical universities adopted this model and organized 1-week workshops for medical students on how to select a research topic, how to write a research proposal, and how to conduct a research study. These universities also established student research centers as a place for peer support and the exchange of ideas, as well as periodic workshops on a variety of topics such as basic research, life skills, etc. [12]. Anecdotal evidence shows that these activities have largely been successful. After 20 years, these centers are still funded and have expanded from one university in 1993 (Tehran University of Medical Sciences) to over 20 universities across the country. The number of Iranian publications indexed in Scopus rose from 1174 in 1998 to 48,809 in 2016; [13] this 42-fold increase may be partly attributed to student trainings that started in the early 1990s.

The ASCEND program is adopting and improving upon the research training model described above. We hypothesize that using this model for undergraduate research training in an HBCU will increase the students’ long-term interest in health research, partly by increasing their research motivation, research competency, sense of self-efficacy, and having a strong science identity [14–16]. The entrepreneurial approach—which gives leading roles in research to students—creates an internal locus of control, which is positively correlated with job satisfaction and performance, [17] as well as self-efficacy [18]. Being involved in the entire research process should also contribute to self-efficacy, which has been associated with commitment to research [8–10].

ASCEND’s model is consistent with scientific findings that some important contributors to happiness at work and the urge to continue are autonomy, sense of purpose (meaning), mastery, a sense of progress, a sense of membership (belonging), and little fear. In her book *Evolve!*, Rosabeth Moss Kanter, a professor at Harvard Business School and chair and director of the Harvard Advanced Leadership Initiative, suggests three primary sources of motivation and initiative in highly innovating companies: meaning, membership, and mastery; [19] all of these elements have been included in the ASCEND model. Choosing the research topic, either individually or via a democratic group process, may lead to a topic that is meaningful to students. Membership and peer support is emphasized in all parts of ASCEND. The ASCEND Scholars work towards mastery as they experience the entire research process, from selecting a topic, to presenting findings to colleagues.

Harvard Business School professor Teresa Amabile and psychologist Steven Kramer reviewed diaries from nearly 12,000 work days, and found that the happiest and most productive days were those marked by a sense of progress [20]. A meta-analysis of data from 420,599 people in over 63 countries suggested that autonomy is a very important predictor of personal happiness and well-being, more so than wealth [21]. Further, in his book *Drive*, Daniel Pink argues that the motivation trifecta is autonomy, mastery, and sense of purpose [22]. These findings from work satisfaction in business are echoed in academia. A national survey shows that faculty at public universities are most satisfied (above all other factors) because their work provides flexibility for determining course content (90.6%) and autonomy and independence (85.8%) [23]. We posit that these factors also will apply to the undergraduates we are training to become self-directed researchers [24].

## ASCEND’s initiatives to enhance student training

**Fig. 1:**
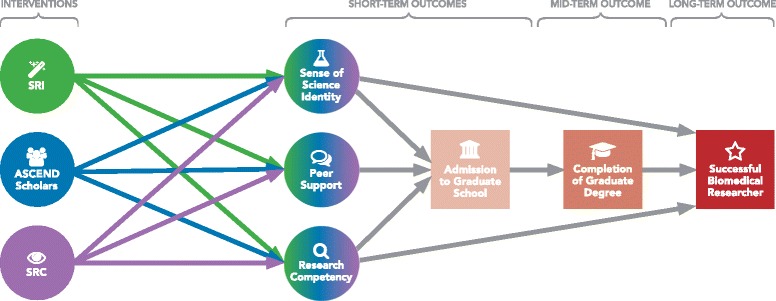
The ASCEND training program’s logic model

ASCEND is implementing three student training initiatives: an annual Summer Research Institute (SRI), a 2-year scholarship program (ASCEND Scholars Program), and the Student Research Center (SRC). All three initiatives are intended to enhance peer support, a sense of science identity, and research competency and self-efficacy. All research training and project topics are health-related and within the mission of NIH. Figure 1 illustrates the ASCEND training program’s logic model.

### The Summer Research Institute (SRI)

The SRI is a research training camp held annually for 25 to 30 students, usually rising sophomores or juniors, from multiple disciplines (e.g., biology, psychology, chemistry, engineering). The main aims of the SRI are to increase participants’ research competency, self-efficacy, peer support, and sense of science identity. Students attending this 8-week workshop-style program familiarize themselves with the concept of health, the different branches of science that contribute to health knowledge, the scientific method, and how scientists think and work. By the end of the program, the students write and receive feedback on a preliminary research proposal. The students learn to work in interdisciplinary teams, and each team is guided by an interdisciplinary team of five faculty members and five near-peer mentors. This interdisciplinary model mimics the real research world [25] and also fosters the development of communities of practice across MSU’s campus [26]. In accordance with the entrepreneurial spirit of ASCEND (as described herein), students select their own research topics and develop their own proposals. We hypothesize that proposing research topics, discussing feasibility of each topic, and writing proposals contributes to greater competency and self-efficacy in research. The SRI faculty and mentors are from MSU, the University of Maryland Medical Center, and Johns Hopkins University. In addition, guest lecturers from these institutions present their work during the SRI.

### The ASCEND Scholars program

The goals of this 2-year training program are to enhance students’ research competency and self-efficacy; enhance their science identity; provide a supportive environment of peers, near-peers (graduate students), and faculty mentors; offer financial support; and offer services that are needed for admission to graduate schools, such as GRE preparation classes. Each year, 20 of the SRI participants who meet the selection criteria are accepted into the ASCEND Scholars Program. During the program, ASCEND Scholars—with the support of mentors—polish the research proposals that they started during the SRI, submit Institutional Review Board (IRB) or Institutional Animal Care and Use Committee (IACUC) applications, and then submit the proposals to ASCEND leadership to be considered for internal funding; if approved, they then conduct their research projects. To mimic real research situations—and to strengthen the sense of entrepreneurship—not all proposals are funded; only those that are considered as competitive at an undergraduate research level are funded. The remaining 5-10 SRI students who are not selected to be ASCEND Scholars will have the opportunity to participate in another MSU research training program such as RISE or choose to become an ASCEND Associate who does not receive a stipend but can participate in many of the ASCEND Scholars program’s activities, such as workshops. Alternatively, they may find that they are not interested in continuing down the research path or may not qualify for any of these programs.

The Scholars also undertake a host of other activities to assist them with their undergraduate studies, and to prepare them for graduate school. Examples of these activities include: working with faculty mentors to prepare Individual Development Plans (IDPs), attending weekly interdisciplinary seminars, traveling to scientific meetings such as the Annual Biomedical Research Conference for Minority Students (ABRCMS), participating in writing seminars, participating in GRE preparation classes (for juniors), and participating in critical thinking workshops. Also, each semester the Scholars are required to register for and attend an ASCEND-specific course. The course has a student-facilitated seminar format, and integrates participants’ research experiences with the science to facilitate understanding of major health care/prevention theories, principles, practices, and procedures. This course is important for its content, but also for the dedicated time it provides students to work with their groups on their research projects, and the opportunity faculty and near-peer mentors have to monitor and support the Scholars.

### The student research center

The SRC is an MSU-registered student organization created to attract, train, and enable its student members to conduct health research, with the aim of fostering students’ leadership skills and increasing their sense of science identity. The underlying assumption is that having an opportunity to run a research center would empower students to think outside of the box, and share their research ideas, as well as identify and acquire relevant training, invaluable peer support, needed resources, and mentorship. This is important as it will allow members to follow their passion, have fun, and take responsibility for their own learning, rather than being passive consumers of knowledge. The SRC is open to all undergraduate full-time students with a grade point average (GPA) of 2.75 or greater. The center is designed to be *of* the students, *for* the students, and *run by* students (except for a staff coordinator and a faculty advisor). The SRC has a dedicated renovated suite (renovations supported by ASCEND) in the science complex that is a student lounge and meeting space with state-of-the-art technology.

## ASCEND’s distinguishing features

ASCEND implements several components that enhance it beyond the traditional programs using the “apprenticeship” training model. One distinguishing feature of ASCEND is its emphasis on students taking the lead in research. For example, during the SRI, each group chooses one of the research topics put forth by each student member and develops a proposal under the guidance of faculty and near-peer mentors. Further, the SRC offers funds for research projects designed and led by students.

A second important feature of ASCEND is that students experience the entire research process; they come up with a topic, write a proposal, apply for ethics approval and funding, conduct the research, analyze data, and disseminate the results. This process should enhance research competency and self-efficacy, and thus help students feel substantially more at ease when they enter graduate school, where they are expected to take the lead and complete their research [27].

A third important feature of ASCEND is its heavy emphasis on group work and peer support. This is manifested in the ASCEND SRI (group work on proposals and group presentations), ASCEND Scholars program (cohorts of 20 students), and the SRC (an environment in which students can learn from each other). Peer support and peer-led teaching may be even more important for minority students, who may have fewer scientists in their families and social networks [28, 29].

A fourth feature is competition. During the SRI, each group of trainees vote to choose a topic from among those suggested by peers; only a subgroup of all proposals developed through the SRI is funded. Likewise, there are research competitions in the SRC, and only some of the proposals will be funded.

Some of the above-mentioned features have been used individually in previous training programs to varying degrees. Collectively, they make ASCEND unique. Most other undergraduate research training programs use the apprenticeship training model: students are placed in a lab where they conduct and master a specific research technique (e.g., running gels), gaining more mastery and independence over time. While the apprenticeship model has been helpful in training many distinguished scientists over the years, one potential shortcoming is that the students may not understand how their efforts contribute to the overall project goals, and some may abandon the idea of pursuing a career in research. For example, a Columbia University student wrote, “One of my friends … an extremely hard-working student with top grades, was looking into both a career in medicine and research. However, his first experience at the bench felt like a burden. He described his time in lab as a series of boring chores with no context and little guidance beyond how to complete the task at hand. Although he later went on to medical school, he abandoned his desire to pursue graduate studies in basic science” [30]. Another potential disadvantage is that this model is very resource intensive, [31] typically requiring one-on-one mentoring, with training outcomes often being critically dependent on the quality of the mentoring environment [32, 33]. Typically, they use less peer support and less competition than what ASCEND offers.

In contrast to “apprenticeship,” we call what ASCEND offers an “entrepreneurship” model for training undergraduate students in biomedical research. In the context of ASCEND, the term “entrepreneurship” has a slightly different meaning than in its business context. In the business context, entrepreneurs are those who have novel ideas, own their idea, take a risk, and develop a product that is intended to be marketed. In the context of ASCEND, students will be entrepreneurial researchers. They will develop their individual research idea (rather than a faculty member’s research idea), own the idea, develop a proposal, and the result will be the students’ research and the knowledge gained.

## Evaluation of the success of the ASCEND training model

ASCEND has included both formative and summative evaluations as an essential part of the program. The summative evaluation is done at both the site level and consortium level.

### Site-level summative evaluation

The long-term measure for the effectiveness of the program is the percentage of students who become successful biomedical researchers and are awarded NIH grants. The intermediate measure is the percentage of students who complete a course of graduate studies in biomedical research. Based on the logic model shown in Fig. 1, short-term measures of success are related to the feasibility of the approach: students’ ability to complete the SRI and write sound research proposals; ASCEND Scholars’ competence to conduct, analyze, and disseminate their proposed studies; and ASCEND Scholars’ adherence to their Individual Development Plans, retention in the program, successful completion of their undergraduate training, and submission of applications for biomedical research graduate degree programs. The site-level evaluation, like the consortium-wide evaluation (described below), will also include measures of science identity for participants of the SRI, ASCEND Scholars, and SRC members. Comparisons will be made with students not enrolled in similar training programs, matched for sex, age, classification, and GPA. If possible, comparisons will be made with students who are enrolled in other training programs (e.g., RISE) and are matched for the same variables mentioned above.

ASCEND evaluators are developing scales and expanding research in the areas related to its mission. For example, ASCEND is developing its own scale to measure “biomedical science identity” among the ASCEND SRI students, ASCEND Scholars, and SRC participants. While there were previous scales to measure science identity, [34] there were no scales optimized for biomedical science identity in college-level students. We have developed and tested one such scale for reliability, which has shown a Cronbach’s alpha of 0.95. Further assessments of reliability and validity of the scale will be done among undergraduate students at MSU, and will be used to compare the above-mentioned groups with appropriate controls (e.g., ASCEND Scholars vs. another group of students matched for age, sex, classification and GPA), or to measure changes over time (e.g., SRI students before and after participation in SRI).

### Consortium-level summative evaluation

The consortium-level evaluation of the BUILD program is conducted by the Coordination and Evaluation Center (CEC) at UCLA. ASCEND provides the CEC with evaluation data in the domains of student progress, faculty development, and institutional development that are aligned with the Hallmarks for Success, which are indicators adopted across the all BUILD sites and the NIH-sponsored Diversity Program Consortium. ASCEND students are compared in terms of individual change on outcomes over time, and based on differences across all BUILD sites, as well as with students at non-BUILD institutions. This situates progress at multiple levels to determine the impact of the ASCEND training model. For more details, please see Davidson et al., this issue [35].

### Formative evaluation

There are very important evaluation activities conducted on a regular basis as part of ASCEND’s continuous quality improvement efforts. For instance, after each SRI module (each week), students provide feedback that is used to determine any changes that can be implemented immediately to improve the students’ learning experience. Likewise, changes to the ASCEND Scholars program are made based on feedback received from students and discussions among faculty and near-peer mentors.

## Sustaining the program by enhancing Morgan State University’s research infrastructure

To sustain resources for student research, MSU needs funds beyond the NIH BUILD grant period. With evidence of success, such funds may be acquired partly through further research grants, partly from MSU’s state budget funds, and partly through funds solicited from philanthropists. Enhancing research funds can be done by improving the faculty’s research competencies, strengthening MSU’s research infrastructure, enhancing a culture of research within MSU, and strengthening collaborations with research-intensive institutions. Fortunately, part of the mission of the BUILD grants, including ASCEND, is to make such enhancements to existing institutional resources.

### Faculty development

ASCEND offers grant writing workshops, pilot research project grants, community-based participatory research grants, funds for travel to conferences, course redesign awards, statistical support, and mentoring workshops. Grant writing workshops are intended to familiarize junior faculty with the NIH grant writing process. ASCEND faculty and staff organize several of these workshops each year, on topics such as the principles of grant writing, writing specific aims for NIH grants, preparing budgets, preparing NIH biosketches, and revising proposals based on reviewer comments.

ASCEND also provides funds for course redesign awards, the purpose of which is to increase students’ interest in biomedical research by encouraging faculty to make their undergraduate health-related courses more interdisciplinary, student-centered, research-training focused, and more appealing to students. Some students can be turned off by “boring” science classes early in their college years, and thus leave STEM majors before they ever get a chance to participate in biomedical research [36]. Research over the past decades has shown that students learn and retain information more effectively if they are actively engaged in constructing their own knowledge [37]. ASCEND funds three to six such curricular change awards every year.

The impact of the ASCEND program’s investment in MSU faculty will be illustrated by metrics such as changes in the number of grant applications, number of grant awards, amount of grant funds, number of publications, etc. We understand that changes in these metrics cannot be totally attributed to ASCEND’s efforts, given the possible effects of multiple other existing variables, such as leadership changes in the university. However, with some effort, we can document which grants or publications directly resulted from ASCEND investments.

### Infrastructure development

ASCEND has renovated three physical spaces in the science complex: two Active Learning Centers and one space for the SRC (described previously). The Active Learning Centers, classrooms with modular furniture, white boards, and other student-centered-learning features, are intended to enhance group work during the SRI and other year-round educational activities.

To increase MSU’s capacity to conduct biomedical research, specifically by providing laboratory training and services for faculty and students, ASCEND has established a Core Laboratory. ASCEND has purchased equipment and is supporting technical staff for the Core Laboratory, which will offer preparatory technology, separations technology, analytics technology, histologic analytics technology, and augmentation of biomedical teaching, such as 3-D human anatomy and virtual microscopy.

### Research collaborations

ASCEND’s local research collaborators include faculty at Johns Hopkins University and the University of Maryland. These research partners review mini-grant proposals submitted to ASCEND, give talks at SRI and SRC seminars, host field trips for SRI students, judge proposals that result from the SRI, work in their labs and on their population-based research studies with the ASCEND Scholars, and provide them with guidance on successfully applying to graduate programs. In addition, they may partner with, or mentor, some of our junior faculty.

ASCEND also plays an important role in cultivating the culture of research, by, for example, increasing the number of MSU faculty who are knowledgeable about NIH (and similar) research grant processes, and creating a community of practice, through which faculty share new pedagogical techniques and research skills, enhancing collaborations with research-intensive institutions. ASCEND has taken several measures to enhance the faculty’s research skills, improve the research infrastructure, and provide additional resources, which are described above. If successful, after several years, the institution will have faculty who are more familiar with research and will be able to secure more research grants, thus having resources to train students.

State funds and philanthropic donations are other sources that can sustain ASCEND’s student-related activities that showed positive impact. The fact that ASCEND’s goals are aligned with MSU’s strategic goals may help in securing such funds. MSU’s five main strategic goals are enhancing student success, enhancing MSU’s status as a doctoral research university, improving and sustaining MSU’s infrastructure, growing MSU’s resources, and engaging with the community. ASCEND’s activities include all of these five areas. In particular, ASCEND will play an important role in student success, and its faculty and institutional activities (e.g., more grant funding and peer-reviewed publications) are consistent with MSU’s long-term strategic plan of enhancing its Carnegie classification [38] of “Doctoral University – Moderate research activity” also known as R3 to an R2 “Doctoral Universities – Higher research activity” institutional mission. This is significant for a public university that began as a teaching institution and that has had (and still has) a student body largely from underserved backgrounds.

## Challenges and solutions

While substantial progress has been made to date, there have been several programmatic challenges. One of these challenges is mentoring, specifically matching mentors to mentees. The students come up with a variety of research topics, and appropriate mentors are usually—but not always—available among MSU or research partner faculty. A solution is to ask faculty who are interested in working with students to lecture during the SRI. Our experiences over the past 2 years show that the topics that some of the students propose are inspired by what they hear from their faculty and near-peer mentors. While the students feel that they own the topic, they also are proposing a research topic for which a good research mentor is available.

Some constructive critics have expressed concern that students’ research projects may not be sufficiently novel. While novelty in research is important, the main purpose of ASCEND is not necessarily to expand the borders of science. ASCEND’s purpose is to train future health researchers, thus we place more emphasis on the learning process than on novelty.

Another challenge is the multidisciplinary nature of the program. Students and faculty come from different disciplines (e.g., public health, biology, chemistry, and psychology). While all of these disciplines can be relevant to health, they use vastly different terminology and research methods, which may make training and group work difficult. Our solution to this problem was that the instructors and near-peer mentors spend some time together before the SRI to discuss issues and harmonize language and terms across disciplines. In addition, the SRI’s curriculum includes important cross-cutting concepts, such as the meaning of health, general research methodology, and proposal writing.

It is not always feasible to find laboratory equipment that students need for research that they propose. However, not all studies require a laboratory; some studies are in the fields of behavioral or public health. When the proposed projects do require a laboratory, the students can use the core laboratory, the laboratory of an MSU researcher, or laboratories of researchers at one of ASCEND’s research partner institutions. We are continuing to expand such opportunities. However, when none of the above exist, the mentors help students modify their research so that the study can be done within the limits of the available equipment.

Time management has been a problem for students. ASCEND Scholars have to spend substantial time in polishing their research proposals, conducting their research projects, and attending a variety of seminars and workshops. If they are members of the ASCEND SRC, they spend time on its activities, too. All these activities could potentially take time away from their other studies, and may impact their GPA, which may negatively affect the success of their graduate school applications. Solutions to date include consolidating some of the activities into a weekly for-credit course, adjusting some of the program requirements, as well as providing students with support through life-skills classes. In all cases, program faculty and staff are responsive to issues as they arise to help the students achieve their goals and experience success at MSU.

## Conclusion

ASCEND initiatives identified as successful by the evaluation can be replicated at other institutions. The overall entrepreneurial model is easy to understand and to apply. The SRI curriculum could be reproduced at other institutions. One of the major concerns about the ASCEND model, raised by a reviewer of the original application, was about its feasibility. It may be difficult for some to believe that undergraduate students would be able to navigate and successfully carry out the complex requirements of designing and implementing robust research studies. ASCEND begins to show how it is possible, with support for motivated first-generation, underrepresented students. Successful implementation of the model along with tested curricula and support systems could pave the way for the implementation of the ASCEND model by other universities nationwide. For example, in a similar setting, a group of student researchers—through their presentations and dissemination of the evidence—played a major role in helping other universities start new SRC chapters [12].

One of ASCEND’s most important contributions is providing a new model for undergraduate research training. The SRI and ASCEND Scholars curricula have so far shown promising results, but more time and further evaluation of their success is needed. The effectiveness of the ASCEND model will be carefully assessed over the next few years, and we will share the results and experiences gained with other institutions.
